# Elevated circulating vascular cell Adhesion Molecule-1 (sVCAM-1) is associated with concurrent depressive symptoms and cerebral white matter Hyperintensities in older adults

**DOI:** 10.1186/s12877-015-0063-7

**Published:** 2015-06-04

**Authors:** Achille E. Tchalla, Gregory A. Wellenius, Farzaneh A. Sorond, Thomas G. Travison, Thierry Dantoine, Lewis A. Lipsitz

**Affiliations:** Institute for Aging Research, Hebrew SeniorLife, Beth Israel Deaconess Medical Center, Harvard Medical School, Boston, Massachusetts USA; Beth Israel Deaconess Medical Center, Boston, Massachusetts USA; Harvard Medical School, Boston, Massachusetts USA; Geriatric Medicine Department, IFR 145 GEIST; EA 6310 HAVAE (Disability, Activity, Aging, Autonomy and Environment), Limoges University, CHU Limoges, Limoges, F-87025 France; Brown University School of Public Health, Providence, Massachusetts USA; Department of Neurology, Stroke Division, Brigham and Women’s Hospital, 45 Francis St, Boston, MA 02115 USA

**Keywords:** Endothelial dysfunction, sVCAM-1, Depression symptoms, Cerebral white matter

## Abstract

**Background:**

Circulating vascular adhesion molecule-1 (sVCAM-1) is a presumed marker of endothelial activation and dysfunction, but little is known about its association with mood. We hypothesized that elevated plasma concentrations of sVCAM-1 may be a marker of depressive symptoms due to cerebral vascular disease.

**Methods:**

We studied 680 community-dwelling participants in the MOBILIZE Boston Study, aged 65 years and older. sICAM-1 and sVCAM-1 were measured by ELISA assay and depressive symptoms were assessed during home interviews using the Revised Center for Epidemiological Studies Depression Scale (CESD-R). Cerebral White Matter Hyperintensities (WMHs) were quantified by MRI in a subgroup of 25 participants.

**Results:**

One hundred seventy nine (27 %) subjects had a CESD-R Score ≥ 16, indicative of depressive symptoms. The mean sVCAM-1 concentration (±SD) was 1176 ± 417 ng/mL in a group with CESD-R Scores <16 and 1239 ± 451 ng/mL in those with CESD-R Scores ≥16 (p = 0.036). CESD-R Score was positively associated with sVCAM-1 (r = 0.11, p = 0.004). The highest quintile of sVCAM-1, which is indicative of endothelial dysfunction, was significantly associated with depressive symptoms compared to the lowest quintile (OR = 1.97 (1.14-3.57) p = 0.015). In a subset of subjects, sVCAM-1 concentration was positively correlated with cerebral WMHs volume (p = 0.018).

**Conclusions:**

The association between high levels of sVCAM-1 and depressive symptoms may be due to endothelial dysfunction from cerebral microvascular damage. Future longitudinal studies are needed to determine whether sVCAM-1 can serve as a biomarker for cerebrovascular causes of depression.

## Backgound

Aging is associated with depressive symptoms, which are an important risk factor for cardiovascular morbidity and mortality [1]. National data from households of adults in the United States show that two-thirds of all those aged 50 years and older who had depression symptoms had a diagnosis of heart disease, stroke, hypertension, and/or diabetes [2]. Depressive symptoms have been also associated with a higher risk of cardiovascular death [3, 4]; and decreased quality of life [5].

Circulating Vascular Cell Adhesion molecule-1 (sVCAM-1) and Intercellular Adhesion Molecule-1 (sICAM-1) are well-known biomarkers of endothelial activation and dysfunction and are associated with an increased risk of hypertension and atherosclerosis [6, 7]. In human studies, increased concentrations of circulating soluble adhesion molecules (CAMs) have been reported in patients with systemic inflammatory and cardiovascular diseases [8, 9]. Increased levels of soluble CAMs in patients with hyperlipidemia may be a marker for atherosclerosis [10]. Increased concentrations of CAMs have also been associated with multiple organ dysfunction, disease severity, or death [11].

It has been hypothesized that late-life depression may be due in part to cerebrovascular disease [12, 13]. Ischemia has been shown to induce the expression of ICAM-1 [14]. In post-mortem studies there is an increased expression of CAMs in the dorsolateral prefrontal cortex in people with depressive symptoms [15], which is consistent with a theory of vascular depression [16, 17]. In addition, there is a correlation between sICAM-1 and depressive symptoms during treatment of melanoma with interferon [18].

We hypothesized that elevated plasma concentrations of circulating CAMs associated with aging may be a marker of depressive symptoms due to cerebral vascular disease. We therefore used plasma biomarkers and data from the MOBILIZE Boston Study (Maintenance Of Balance, Independent Living, Intellect, and Zest in the Elderly) to explore the relationships between plasma levels of CAMs and depressive symptoms in a community-based population of older adults.

## Materials and Methods

### Participants

The study sample consisted of 680 community-dwelling seniors living in the Boston area who participated in the MOBILIZE Boston Study (MBS). The design and methodology for this study have been previously described in detail [19, 20]. In brief, 765 persons were enrolled using door-to-door population based recruitment. To be included, individuals had to be > 65 years, able to understand and communicate in English, able to walk 20 feet without personal assistance (walking aids permitted), and expected to live in the area for at least 2 years. Exclusion criteria included terminal disease, severe vision or hearing deficits, and Mini-Mental State Examination score <18 [21, 22]. All subjects underwent a complete home and laboratory assessment of demographic characteristics, medical conditions, medications, functional status, gait speed, smoking status, alcohol use, blood pressure, and cerebral hemodynamics at baseline.

### Depressive Symptoms Assessment

Depressive symptoms were assessed at study enrollment using the Revised Center for Epidemiologic Studies Depression scale (CESD-R) [23]. The CESD-R is a 20-item self-administered instrument designed to measure the presence of depressive symptoms over the previous two weeks in community studies. Note that the CESD-R does not capture information on a patient's clinical or treatment history and is not useful as a diagnostic tool for depression. The CESD-R has been validated [24, 25]. As in previous work [26], we chose *a priori* to use scores of <16 to indicate no or minimal depressive symptoms and ≥16 to indicate the presence of moderate or severe symptoms.

### Biomarker measures

Plasma concentrations of sICAM-1, sVCAM-1, and interleukin-6 (IL-6) were measured by ELISA assay (R&D Systems, Minneapolis, MN). For sICAM-1 this assay has a sensitivity of 0.35 ng/mL, and the day-to-day variability of the assay at concentrations of 64.2, 117, 290 and 453 ng/mL are 10.1, 7.4, 6.0 and 6.1 %, respectively. For sVCAM-1 the assay has a sensitivity of 2.0 ng/mL and the day-to-day variability of the assay at concentrations of 9.8, 24.9 and 49.6 ng/mL is 10.2, 8.5 and 8.9 %, respectively. For IL-6, the assay has a sensitivity of 0.094 pg/mL, and the day-to-day variability of the assay at concentrations of 0.49, 2.78 and 5.65 pg/mL are 9.6, 7.2 and 6.5 %, respectively. In addition, the concentration of high sensitivity C-reactive protein (hsCRP) was determined using an immunoturbidimetric assay on a Hitachi 917 analyzer (Roche Diagnostics - Indianapolis, IN), using reagents and calibrators from DiaSorin (Stillwater, MN). This high-sensitivity assay has a limit of detection of 0.03 mg/L. The day-to-day variability of the assay at concentrations of 0.91, 3.07 and 13.38 mg/L are 2.81, 1.61 and 1.1 %, respectively. All assays were performed by Dr. Nader Rifai’s group at Boston Children’s Hospital.

### Magnetic Resonance Image data

Volumes of cerebral white matter hyperintensities (WMH) normalized by intracranial volume were estimated from the MRI data in a subset of 25 MBS participants who completed MRI substudy. Eligible and willing participants were imaged using a Siemens Trio 3 Tesla system (Erlangen, Germany) employing a 12-channel phased-array head coil for reception and body coil for transmission. Cerebral WMH were measured with Free Surfer (http://surfer.nmr.mgh.harvard.edu) using a multispectral procedure that classifies white matter as normal or abnormal from signal intensities from the T1, PD, and T2 images at each voxel. The Free Surfer procedure for lesion segmentation combines the initial standard segmentation with an extension of the subcortical segmentation procedure that incorporates information from a co registered T2 and PD image for the segmentation of signal abnormalities within the white matter. Although the procedure with T1-weighted images alone tends to underestimate white matter lesion volumes, the incorporation of information from T2/PD provides more robust estimation of the lesion volumes. The multiple image modalities were registered to the T1 with boundary-based registration (BBR) and the segmentation of WMH from healthy WM was accomplished with a multispectral Gaussian classifier for each subject based on the atlas values.

### Other covariates

Covariates included sociodemographic characteristics, health status, and amount of physical activity. Sociodemographic characteristics assessed in the home interview included age, sex, race (self-identified), and years of education. We used the validated Physical Activity Scale for the Elderly (PASE) to measure physical activity in the previous week [27]. Participants were asked about physician-diagnosed major medical conditions. Details of the study variables have been published previously [19, 20]. Diabetes was defined using an algorithm based on self-reported diabetes, use of antidiabetic medications, and laboratory measures from the baseline clinic visit, including random glucose (≥200 mg/dL) and hemoglobin A_1c_ (≥7 %). Body mass index (calculated as weight in kilograms divided by height in meters squared) was calculated from measured height and weight. Comorbidity index was the number of comorbidities or medical conditions. Medication use (antihypertensive, antidepressant, and benzodiazepine) was also assessed.

### Ethics Statement

The MOBILIZE Boston Study was reviewed and approved by the Hebrew SeniorLife Institutional Review Board (IRB). Written informed consent was obtained from each participant. The study was conducted according to the principles of the Helsinki Declaration.

### Data Analysis

Concentrations of sVCAM-1 and sICAM-1 were natural log-transformed to approximate a normal distribution prior to modeling as continuous variables. We also divided the distributions of sVCAM-1 and sICAM-1 into quintiles according to the distribution in the entire study population for categorical analyses. We compared baseline characteristics of different groups of study participants by using *t* tests, χ2 tests, or Wilcoxon rank-sum tests, as appropriate.

We used multivariate logistic regression to estimate the odds ratio and 95 % confidence intervals (CIs) for quintiles of sVCAM-1 and sICAM-1 and depressive symptoms (CESD-R Score ≥16). Analyses were adjust for the following groups of confounders: 1) other biomarkers (IL6, C-Reactive Protein); 2) socio-demographic conditions (age, gender, white race, education level, BMI, current smoker, alcohol use); 3) Health conditions (diabetes, hypertension, congestive heart failure, hyperlipidemia, cognitive status, any cardiovascular medications, coronary disease, previous stroke), and 4) physical activity level.

Subjects with missing data for depressive symptoms, sVCAM-1 and sICAM-1 were excluded. All analyses were performed using SAS software, version 9.3 (SAS Institute Inc, Cary, North Carolina). A two-sided P value of less than 0.05 was considered indicative of statistical significance.

## Results

### 1. Subject Characteristics

As shown in Table 1, 489 (73.2 %) participants had no depression symptoms and 179 (26.8 %) had depressive symptoms. The mean IL6 was significantly higher in those with depression symptoms. 605 (90.6 %) had a least one cardiovascular disease, those with depression had significantly more co-morbidities (p < 0.0001) and performed less physical activity (p = 0.007) than those without.

**Table 1:** Characteristics of participants according to CESD-R score status (<16 vs. ≥ 16), N = 668*

Baseline Characteristics	CESD-R score^a^		*P* Value^‡^
	<16 (n = 489, 73 %)	≥16 (n = 179, 26 %)	
**Demographics**			
Age, mean (SD), y	77.9 ± 5.3	78.4 ± 5.5	0.44
Gender			0.071
Men	193 (39.5)	57 (31.8)	
Women	296 (60.5)	122 (68.2)	
White Race	397 (81.2)	138 (77.0)	0.58
Educational level, mean (SD), y	14.98 ± 5.1	14.97 ± 8.1	0.11
**Health behaviors**			
Body mass index, kg/m2,^b^			0.31
<25	148 (30.3)	62 (34.6)	
25-29.9	208 (42.5)	78 (43.6)	
≥30	133 (27.2)	39 (21.8)	
Current smoker	283 (57.9)	102 (57.)	0.84
Alcohol use (Endorsing ≥2 drinks per week)	136 (27.8)	35 (19.6)	0.03
Physical activity score^c^			0.024
0- 66	139 (28.4)	68 (38.0)	
66.01-124	164 (33.5)	59 (33.0)	
124.01-559	181 (37.0)	49 (27.4)	
**Health conditions**			
Comorbidity index	2.8 ± 1.4	3.8 ± 1.8	<.0001
Hypertension	374 (76.5)	147 (82.1)	0.075
Hyperlipidemia	285 (58.3)	103 (57.5)	0.54
Diabetes	84 (17.2	42 (23.5)	0.066
Previous Stroke	39 (8.0)	27 (15.1)	0.007
Coronary artery disease	67 (13.7)	40 (22.4)	0.0044
Congestive Heart failure	16 (3.3)	18 (10.1)	0.0004
Cognitive impairment (MMSE < 24)^d^	42 (8.6)	30 (16.8)	0.0026
**Medications**			
Any cardiovascular medication	320 (65.4)	134 (74.86)	0.024
Psychotropic Medication	30 (6.1)	16 (8.94)	0.21
**Biomarkers measures**			
C-Reactive Protein, mean (SD), mg/L	3.8 ± 12.3	5.3 ± 14.8	0.19
Interleukin-6, mean (SD), pg/mL	3.8 ± 7.5	4.2 ± 5.7	0.046
Soluble ICAM-1, mean (SD), ng/mL	258 ± 75	273 ± 87	0.064
Soluble VCAM-1, mean (SD), ng/mL	1176 ± 417	1239 ± 451	0.036

*sVCAM-1* Soluble Vascular Cell Adhesion Molecule-1, *sICAM-1* Soluble Inter Cellular Adhesion Molecule-1. *Missing values = 12 from 680;‡Global test: χ2 or Fisher’s exact test for binary variables; analysis of variance for continuous variables.

^a^CESD-R, Center for Epidemiological Studies Depression Scale Revised

^b^Body mass index is calculated as weight in kilograms divided by height in meters squared

^c^Physical activity tertiles measured using the Physical Activity Scale for the Elderly

^d^Mini-Mental State Examination (MMSE) cut off point for cognitive impairment

Although IL6 was higher in the group with depressive symptoms, it was not linearly correlated with CESD-R score (r = 0.03 p = 0.32).

### 2. Soluble VCAM-1 and Depression symptoms (CESD-R ≥16)

The mean sVCAM-1 concentration was 1177. ± 417 in the group without depressive symptoms (CESD-R < 16) and 1239 ± 451 in the group with depressive symptoms (CESD-R ≥ 16) (p = 0.036). Univariate logistic regression analyses showed associations between sVCAM-1 and many cardiovascular diseases, comorbidity index, and less physical activity (Table 2). The unadjusted model (Model 1) showed that the highest quintile of sVCAM-1 was associated with depression symptoms (CESD-R ≥16) (OR = 2.28 (1.24 – 3.84) p = 0.0066). After adjustment, the final multivariate logistic regression model (Model 4) showed that the highest quintile of sVCAM-1 compared to lowest quintile was significantly associated with depressive symptoms (CESD-R ≥16) (OR = 1.97 (1.14 – 3.57) p = 0.015) (Table 3).

**Table 2:** Univariate logistic regression model with CESD-R score status ( <16 vs. ≥ 16)^a^, N = 668*

Baseline Characteristics	OR	95%CI	*P* Value
Age, mean (SD), y	1.1	(0.99, 1.05)	0.30
Gender			
Men	1.0	Reference	
Women	1.4	(0.97, 2.01)	0.072
Race			
Other race	1.0	Reference	
White Race	0.8	(0.49, 1.28)	0.34
Body mass index, kg/m2,^b^			
<25	1.18	(0.75, 1.66)	0.58
25-29.9	1.00	Reference	
≥30	0.78	(0.50, 1.22)	0.27
Current smoker	0.96	(0.68, 1.36)	0.84
Alcohol use (Endorsing ≥2 drinks per week)	0.63	(0.42, 0.96)	0.031
Physical activity score^c^			
0- 66	1.81	(1.18, 2.77)	0.007
66.01-124	1.33	(0.86, 2.05)	0.20
124.01-559	1.00	Reference	
Comorbidity index	1.49	(1.32, 167)	<.0001
Hypertension	1.50	(0.96, 2.36)	0.076
Hyperlipidemia	1.06	(0.74, 1.54)	0.74
Diabetes	1.48	(0.97, 2.25)	0.067
Previous Stroke	2.05	(1.21, 3.45)	0.0075
Cognitive impairment (MMSE < 24)^d^	2.14	(1.30, 3.55)	0.003
Congestive Heart failure	3.33	(1.66, 6.67)	0.0007
Coronary artery disease	1.23	(1.07, 1.43)	0.0048
Any cardiovascular medication	1.56	(1.06, 2.30)	0.025
Psychotropic Medication	1.50	(0.79, 2.81)	0.21

*Missing values = 12 from 680.

^a^CESD-R, Center for Epidemiological Studies Depression Scale Revised

^b^Body mass index is calculated as weight in kilograms divided by height in meters squared

^c^Physical activity tertiles measured using the Physical Activity Scale for the Elderly

^d^Mini-Mental State Examination (MMSE) cut off point for cognitive impairment

**Table 3:** Odds ratio from logistic regression relationship between levels of soluble VCAM-1 and depression symptoms (CESD-R score ≥16), No. = 668†

	Quintiles of sVCAM-1^‡^, Median (IQR), ng/mL					*P* for trend
	736 (660–810)	937 (899–970)	1115 (1071–1169)	1332 (1277–1389)	1711 (1567–2011)	
No.	132	135	135	132	134	
Model 1						
Odds ratio (95 % CI)	1.00	1.58 (0.88-2.82)	1.68 (0.94-2.97)	1.59 (0.89-2.83)	2.18 (1.24-3.84)	
*P* value	Reference	0.13	0.08	0.12	0.0066	0.011
Model 2						
Odds ratio (95 % CI)	1.00	1.59 (0.89-2.86)	1.61 (0.90-2.87)	1.53 (0.85-2.74)	1.96 (1.09-3.51)	
*P* value	Reference	0.12	0.10	0.15	0.024	0.025
Model 3						
Odds ratio (95 % CI)	1.00	1.69 (0.93-3.08)	1.62 (0.90-2.92)	1.55 (0.85-2.82)	2.10 (1.17-3.78)	
*P* value	Reference	0.12	0.11	0.15	0.024	0.026
Model 4						
Odds ratio (95 % CI)	1.00	1.79 (0.95-3.39)	1.65 (0.88-3.07)	1.68 (0.89-3.17)	1.97 (1.14-3.57)	
*P* value	Reference	0.07	0.12	0.11	0.015	0.030

^‡^ *sVCAM-1*: Soluble Vascular Cell Adhesion Molecule-1; CI, confidence interval; IQR, interquartile range.

†Missing values = 12 from 680; Model 1: non adjusted; Model 2: adjusted for Biomarkers ( IL6, C-Reactive Protein); Model 3: additionally adjusted for Socio-demographic condition (Age, gender, White race, Body Mass Index, Current Smoker, Alcohol use, physical activity); Model 4: additionally adjusted for Health conditions (Diabetes, Hypertension, Congestive Heart failure, Cognitive status, Any cardiovascular medications, Coronary disease, Previous stroke, any cardiovascular medication and psychotropic medication)

### 3. Soluble ICAM-1 and other biomarkers

sICAM-1 and hs-CRP concentrations were both weakly correlated with CESD-R score (respectively, r = 0.12 p = 0.0028 and r = 0.08 p = 0.046. After adjustment for sVCAM-1, age, gender, white race health condition and physical activity score, the final model showed no significant relationships between depressive symptoms and these biomarkers.

### 4. Cerebral WMHs, sVCAM-1, and Depression symptoms

Table 4 summarizes the characteristics of the 25 participants with brain MRI data.

**Table 4:** Demographics and clinical characteristics of participants with MRIs at baseline, No =25

Baseline Characteristics	MRI data
**Demographics**	
Age, mean (SD), y	77.6 ± 6.3
Women	17 (68.0)
White Race	23 (92.0)
Educational level, median (IQR), y	16 [14–17]
**Medical condition**	
Comorbidity index, mean (SD)	2.7 ± 1.9
Cardiovascular disease	24 (96.0)
**Medication**	
Any psychotropic medication	3 (12.0)
Any cardiovascular medication	16 (64.0)
**Brain imaging**	
White matter hyperintensities volume, mean (SD), mL	15.84 ± 12.61
White matter hyperintensities % ICC, mean (SD)	1.08 ± 0.86
**Biomarkers measures**	
C-Reactive Protein, Mean (SEM),mg/L	1.6 ± 0.5
Interleukin-6, Mean (SEM),pg/mL	2.7 ± 0.6
Soluble ICAM-1, Mean (SEM), ng/mL	268 ± 19
Soluble VCAM-1, Mean (SEM), ng/mL	1172 ± 96

*sVCAM-1*: Soluble Vascular Cell Adhesion Molecule-1; *sICAM-1*: Soluble Inter Cellular Adhesion Molecule-1. *Missing values = 12 from 680

**Fig. 1:**
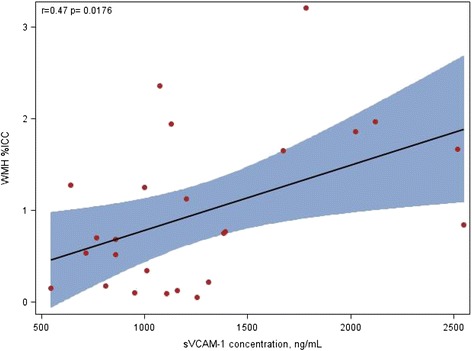
The linear correlation between Cerebral White Matter Hyperintensities (WMHs) as a percentage of Intracranial Cavity Capacity (% ICC) and Soluble Vascular Adhesion Molecule-1 (sVCAM-1) concentration in ng/mL, No. =25 subjects

We observed that cerebral WMH volume was correlated with sVCAM-1 concentration (r = 0.47 p = 0.018) and the association still significant when adjusted for comorbidity index (Fig 1).

WMHs volumes were also higher among participants with CESD-R ≥ 16; 22.43 mL vs. 10.78 mL (p = 0.039) but not reach statistical significance when adjusted for comorbidity index (p = 0.056).

## Discussion

The results of this study showed cross-sectional associations between elevated plasma levels of sVCAM-1 and 1) depressive symptoms, and 2) cerebral white matter damage among older community-dwelling adults.

Research suggests that mood can become impaired when one or more of the brain’s frontal-subcortical circuits are damaged [28, 29]. Three of these circuits (dorsolateral prefrontal, lateral orbitofrontal, and medial frontal/anterior cingulate) play an important role in mood regulation, and damage in these areas produces a neurobehavioral syndrome. Previous studies suggested that a relationships between white matter hyperintensities, cerebral blood flow regulations [30, 31]. Brain endothelial dysfunction indicated by higher levels of sVCAM-1 may be a key pathogenic mechanism. Elevated levels of plasma sVCAM-1 concentration (>1200 ng/mL) may signal vascular damage in the cerebral white matter that carries axons from frontal-subcortical circuits involved in mood regulation. Furthermore, endothelial dysfunction may impair cerebral blood flow regulation, resulting in ischemic damage to these circuits [32, 33].

Previous work by our group and others [34] has shown that elevations in sVCAM-1 are associated with abnormal CO_2_ vasoreactivity in the brain. Those with depressive symptoms seem to have higher WMHs volumes, but in our small subsample with MRI data, this association did not reach statistical significance after adjustment for comorbidities index.

This current study had some limitations: first, its cross-sectional design precludes investigating the temporal relation between CAMs and depressive symptoms. The second is that our population may be not representative of all older adults. We could only examine the relationship between sVCAM-1 and WMHs in a small subsample that was willing and able to undergo MRI studies, which may be not representative of the general population. The third is that we did not examine other mechanisms of depressive symptoms. The link between depressive symptoms and sVCAM-1 may be attributable, in part, to a common genetic vulnerability or to pathophysiologic factors, including increased platelet reactivity, an underlying inflammatory state, or situational stresses that may be associated with elevated sVCAM-1 plasma levels [35, 36]. We tried to address this in part, by controlling for the inflammatory biomarkers CRP and IL6 in the multivariable analysis and our results suggest that plasma levels of sVCAM-1 was independently associated with depression symptoms.

## Conclusion

In summary, our study suggests that elevated plasma levels of sVCAM-1 may be a biomarker for the presence of cerebral microvascular disease in community-dwelling elderly people with symptoms of depression. It may be important to identify elderly people with high levels of sVCAM-1 as having a high risk of late life depression, and to treat their atherosclerotic and cardiovascular disease rigorously for secondary prevention. Additional prospective studies are needed to confirm our findings and determine whether sVCAM-1 can serve as a biomarker for cerebrovascular causes of depression.
